# DefiGame, a serious game to discover neurodevelopmental disorders

**DOI:** 10.1192/j.eurpsy.2024.56

**Published:** 2024-08-27

**Authors:** C. Bonnet, C. Immesoete, Specialists and parents affiliated to the french rare disease network DéfiScience - Professeur Vincent des Portes, Mme Marie-Pierre Reymond, Mme Caroline Immesoete, Mme Fanny Forel, Mme ANTOINE Odile, Mme AUPETIT Laure, Mme BENOIT Céline, Docteur BROSSEA

**Affiliations:** Filière DéfiScience, LYON, France

## Abstract

**Abstract:**

Défigame is a training tool designed in collaboration with parents and specialists from the French Rare Diseases Network DéfiScience.

In this serious game, you take on the role of a general practitioner treating four young patients whose developmental trajectories raise questions. Interactively and with the help of concrete tools, you’ll learn about the recommendations for coordinating an appropriate course of prescription, care and support for a family, from the search for a diagnosis to early management of a Neurodevelopmental Disorder (NDD).

WHO IS DEFIGAME FOR?

1. Any European doctor questioning the etiology of a neurodevelopmental disorder, prescribing genetic tests or wishing to update their knowledge in the field of NDD, particularly in relation to a rare disease.
2. Any other healthcare professional concerned with etiological diagnosis or support for people with NDD and their families.

**Disclosure of Interest:**

None Declared

